# Rapid and accurate detection of RMP- and INH- resistant *Mycobacterium tuberculosis* in spinal tuberculosis specimens by CapitalBio™ DNA microarray: A prospective validation study

**DOI:** 10.1186/1471-2334-12-303

**Published:** 2012-11-14

**Authors:** Zehua Zhang, Litao Li, Fei Luo, Peng Cheng, Feng Wu, Zheng Wu, Tianyong Hou, Min Zhong, Jianzhong Xu

**Affiliations:** 1 Department of Orthopaedics, Southwest Hospital, Third Military Medical University, Chongqing, China; 2 Department of Orthopaedics, The 309th Hospital of PLA, Beijing, China; 3 Institution of Pathology, Southwest Hospital, Third Military Medical University, Chongqing, China; 4 Department of Clinical Laboratory, Infectious Disease Medical Center, Chongqing, China

**Keywords:** DNA microarray, Spinal tuberculosis, Drug resistance, Gene mutation

## Abstract

**Background:**

DNA microarrays can detect tuberculosis and its multi-drug resistant form in *M. tuberculosis* isolates and sputum specimens with high sensitivity and specificity. However, no performance data currently exists for its use in spinal tuberculosis specimens. This study was aimed to assess the performance of the CapitalBio™ DNA microarray in the detection of isoniazid (INH) and rifampicin (RMP) resistance in spinal tuberculosis compared with the BACT/MGIT 960 system.

**Methods:**

From March 2009 to December 2011, 153 consecutive patients from Southwest Hospital, Chongqing with clinically and pathologically diagnosed spinal tuberculosis were enrolled into this study. Specimens collected during surgery from the tuberculosis patients were subjected to *M. tuberculosis* species identification and drug-resistance detection by the CapitalBio™ DNA microarray, and results were compared with those obtained from the absolute concentration drug susceptibility testing.

**Results:**

The CapitalBio™ DNA microarray achieved 93.55% sensitivity for the correct *M. tuberculosis* species identification of the 93 specimens that tested positive for spinal tuberculosis through culture. In addition, twenty-seven additional patients (45.0%) were detected by the DNA microarray to be positive for *M. tuberculosis* among sixty spinal tuberculosis patients who were culture negative. Moreover, the DNA microarray had a sensitivity of 88.9% and a specificity of 90.7% for RMP resistance, and the microarray had a sensitivity of 80.0% and a specificity of 91.0% for INH resistance. The mean turn-around time of *M. tuberculosis* species identification and drug resistance detection using the DNA microarray was 5.8 (range, 4–9) hours.

**Conclusions:**

The CapitalBio™ DNA microarray is a feasible and accurate tool for the species identification of *M. tuberculosis* and for directly detecting RMP and INH resistance from spinal tuberculosis specimens in fewer than 9 hours.

## Background

Multi-drug resistant tuberculosis (MDR-TB) is becoming a serious public-health concern in developing countries. The World Health Organization (WHO) has estimated that there are 440,000 incident MDR-TB cases, and 150,000 persons with MDR-TB died worldwide in 2008 [1]. China is currently ranked 1^st^ among the 27 countries with the highest burden of MDR-TB and extensively drug-resistant tuberculosis (XDR-TB) in the world [1]. As the most common extrapulmonary tuberculosis, spinal tuberculosis can lead to severe complications and is also facing the problem of drug resistance. The emergence of drug-resistant spinal tuberculosis may lead to an increased number of relapses and initial treatment failures [2]. Therefore, early diagnosis of drug resistance is essential to the optimal management of drug-resistant spinal tuberculosis.

Conventional drug susceptibility testing (DST) can provide definitive results, but this approach is time-consuming and usually requires several weeks to produce susceptibility profiles, can lead to inadequate treatment and further acquired resistance during this period [3]. Moreover, DST for tuberculosis spondylitis is not performed routinely in most resource-poor hospitals in China because of the biosafety concerns and inadequate infrastructure, which present a major hindrance for the treatment of the disease. Therefore, there is an urgent need to develop accurate, rapid and feasible molecular methods to detect the drug-resistance.

Recently, a multitude of commercial molecular DST kits, such as INNO-LiPA, Genotype MDR-TBplus, CapitalBio™ DNA microarray and Xpert MTB/RIF, have been developed for the detection of mutations associated with resistance to RMP and INH [4-12]. Among them, the CapitalBio™ DNA microarray that incorporate specific nucleotides at a given position of the *rpoB*, *inhA* and *katG* gene has been developed by CapitalBio Corporation to detect tuberculosis and its multi-drug resistant form in *M. tuberculosis* isolates and sputum specimens with notable sensitivity and specificity [6,11,12]. Previous study reported that the system had an accuracy of 91.8% in RMP susceptibility prediction and 70.2% in INH, compared with phenotypic DST [11]. However, there is no data to support this technique’s application for testing spinal tuberculosis specimens. In our study, we aimed to access the feasibility and accuracy of the CapitalBio™ DNA microarray for parallel species identification and detection of mutations that confer INH and RMP resistance among consecutive spinal tuberculosis patients.

## Materials and methods

### Study design

From March 2009 through December 2011, we performed this prospective validation study in Southwest Hospital, Chongqing, China. One hundred and fifty-three consecutive patients with clinically and pathologically diagnosed spinal tuberculosis were enrolled into this study. Clinical specimens (e.g., pus, caseous or granulation tissue) were collected during surgery and were divided into two aliquots for tuberculosis detection; one of the aliquots was subjected to species identification and detection of gene mutations by the CapitalBio™ DNA microarray (CapitalBio Corp., Beijing, China), and the other aliquot was processed for culture and DST. The study was approved by the Ethics Committee of Southwest Hospital. Written informed consent was obtained from all of the patients.

### Diagnostic criteria for the spinal tuberculosis

Diagnosis of spinal tuberculosis was made with reference to clinical and radiological findings and was verified histopathologically after debridement in all patients. Clinical and laboratorial presentation: Fever, night sweat and weight loss, back pain, neurological signs, angular deformity, positive response to anti-tuberculosis treatment, relative lymphocytosis, a low level of haemoglobin and a raised ESR. Radiology: X-ray, CT and MRI revealed bone destruction, vertebral collapse, kyphosis, tubercular lesions and paravertebral abscess. Histopathology: Epitheloid granulomas, a granular necrotic background and lymphocytic infiltration.

### CapitalBio™ DNA microarray design and preparation

**Figure 1:**
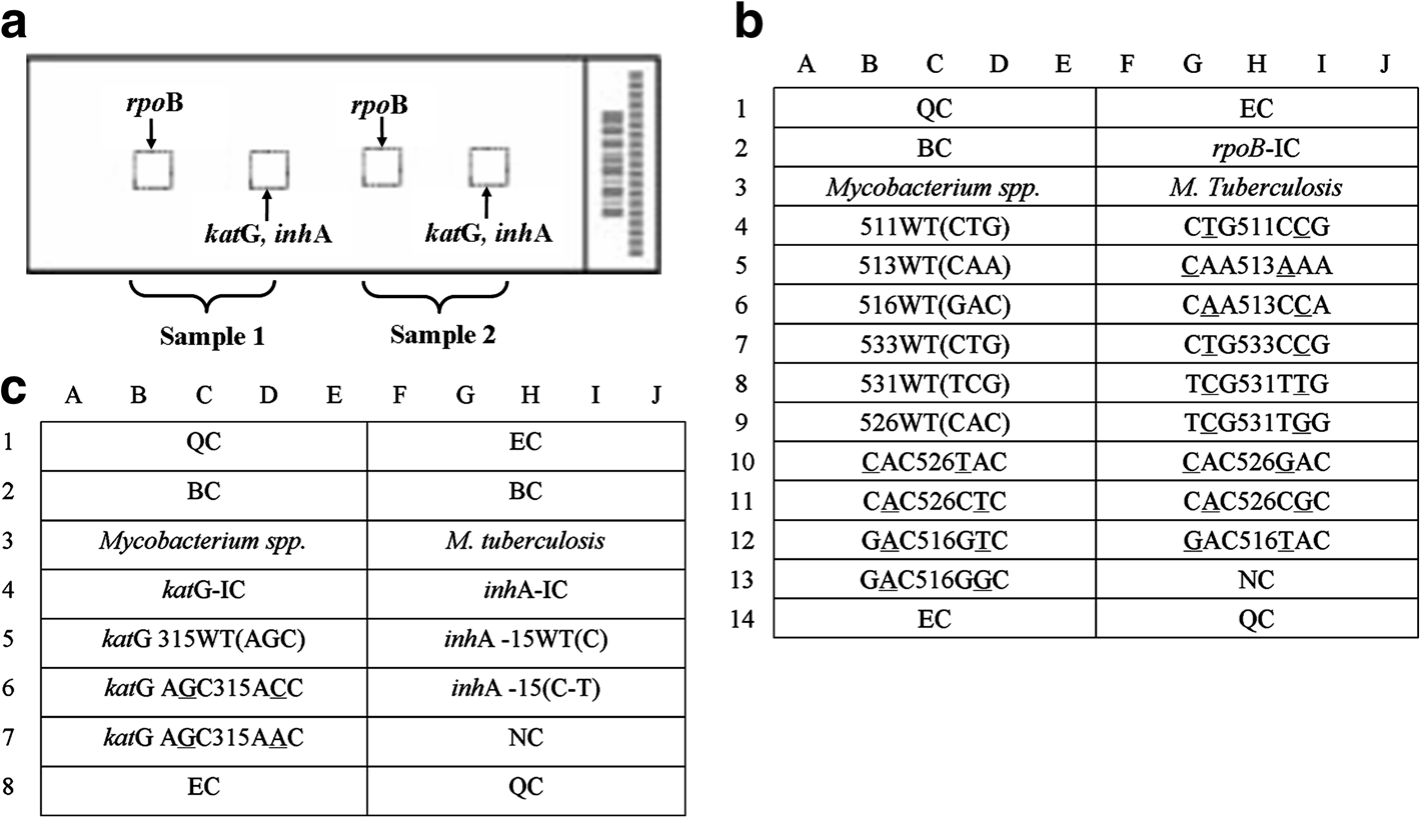
**Schematic diagram of the DNA probe array for *rpoB *,*katG *and *inhA *detection. a**. The biochip contains two microarrays and two specimens can be analyzed in parallel; for each array, one sub-array is for RMP, and the other is for INH. **b**. Six *rpoB* wild-type probes and thirteen mutation-type probes were designed for the detection of RMP resistance. **c**. For the detection of INH resistance, one probe covers the wild-type codon 315 of *katG* and two mutation-type probes for the same region, while one wild-type probe and one mutation-type probe detect the *inhA* promoter region. All probes were immobilized horizontally for five times. QC: quality controls, EC: external controls, BC: blank controls, NC: negative controls, IC: internal controls, WT: wild type.

**Figure 2:**
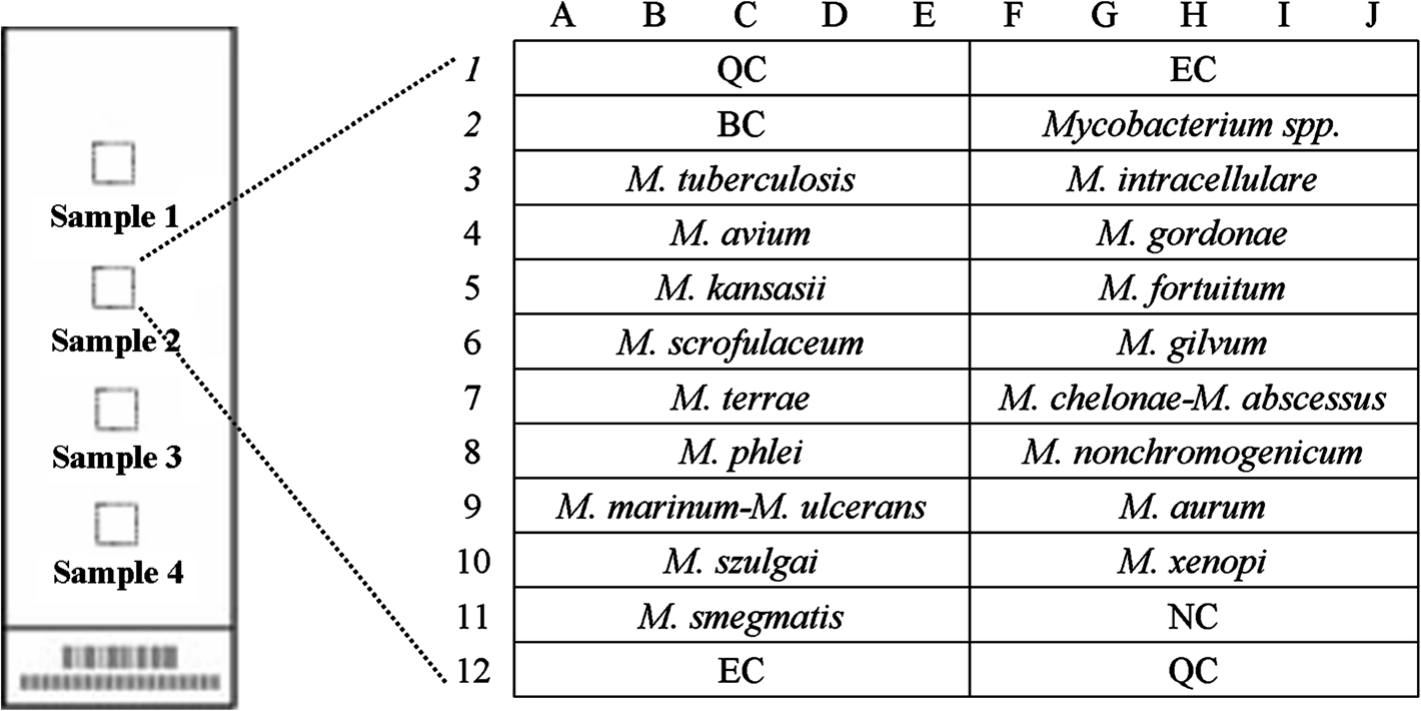
**Schematic diagram of the DNA probe array for different *Mycobacterium *specimens.** Seventeen oligonucleotide probes were chosen in several species-specific sequence regions of the 16S rRNA gene for identification of different Mycobacterium species. All probes were immobilized horizontally five times.

Oligonucleotide probes were printed onto OPAldehydeslide aldehyde-activated slides at a concentration of 10 μmol/L in 50% dimethyl sulfoxide using a SmartArrayer-48 microarrayer (both from CapitalBio Corp., Beijing, China) and were covalently immobilized on the slides via an amino group at their 5’ ends (spotting pattern shown in Figure 1). In each array, sixteen oligonucleotide probes were designed to detect mutations of *rpoB* (codons 531, 526, 513, 516, 511, 533), *inhA* (nucleotide-15 within the promoter) and *katG* (codon 315) (Figure 1 a, b, c). In addition, seventeen oligonucleotide probes were chosen in several species-specific sequence regions of the 16S rRNA gene for identification of different *Mycobacterium* species (Figure 2).

### Assay Procedure for the CapitalBio™ DNA microarray

Spinal tuberculosis specimens were subjected to DNA microarray analysis at the Laboratory Department of Southwest Hospital. Investigators and laboratory staff were blinded to the culture and DST results of the clinical specimens. The CapitalBio™ DNA microarray was performed according to the manufacturer’s instructions [11,12]. Briefly, 1–2 ml of the spinal tuberculosis specimen was inactivated for 30 min at 95°C followed by sonication in an ultrasonic water bath for 25 min. Next, the inactivated specimen was liquefied with an equal volume of liquefying solution (containing 0.5% N-acetylcysteine, 1.45% trisodium citrate and 2% NaOH) for 30 min at 37°C. After inactivation and liquefaction, 1 ml of the specimen was centrifuged at 12000 rpm for 5 min to pellet the bacteria. Following centrifugation, the supernatant was discarded, and the pellet was resuspended in 1 ml of 0.9% (w/v) saline and centrifuged at 12000 rpm for 5 min. This supernatant was subsequently discarded, and the pellet was resuspended in 50 μl of 10 mM Tris-EDTA buffer and then transferred to an extraction tube. The total DNA was isolated from the sample by vortexing the tube at maximum speed in an Extractor™ 36 (CapitalBio) for 5 min. Next, the extraction tube was incubated at 95°C for 5 min, centrifuged briefly and the supernatant was stored at −20°C until use. Next, asymmetric PCR was performed on the samples by a Peltier PTC225 thermal cycler (MJ Research, Watertown, MA) for two amplification rounds according to the manufacturer’s instructions. Following PCR amplification, chip hybridization was performed on the samples in a three-dimensional tilting agitator BioMixer II hybridization; an automated SlideWasher-8 (both from CapitalBio) was then used to wash and dry the hybridized slides. The fluorescent signal on the slides that was emitted by the microarrays was detected using a GeneArray scanner (LuxScan™-10K confocal laser scanner, CapitalBio, China), and the fluorescence intensities were quantified by the tailor-made software developed by CapitalBio. The whole protocol took less than 9 h to perform, with up to 4 specimens being analyzed for *M. tuberculosis* identification and 2 specimens being analyzed for drug resistance detection in parallel.

### Conventional culture and DST

The quality-assured culture and DST was conducted using the BACT/MGIT 960 system and the absolute concentration method on L-J medium at the Department of Clinical Laboratory, Infectious Disease Medical Center in Chongqing. The specimens were processed according to standard methodologies [13]. The following critical concentrations were used respectively: 1 and 10 μg/ml for INH, and 50 and 250 μg/ml for RMP.

## Results

### Study population

We enrolled a total of 153 patients with spinal tuberculosis at Southwest Hospital during the study period. Among the 153 patients, 76 were female and 77 were male. The mean age was 36.4 years (range, 4 to 76 years). A total of 77.1% (118/153) of the patients were initial treatment cases, and the remaining 22.9% (35/153) were retreatment cases who had received previous chemotherapy for a mean period of 7.1 months (range, 3 to 74 months) at the time of enrollment. None of the patients was HIV-seropositive.

### The results of conventional culture and DST

Among the 153 specimens that were cultured, a total of 60.1% of the specimens (93/153) produced a positive culture. Following culture testing, the positive specimens were subjected to DST. The results of the DST using the absolute concentration method on L-J medium showed that a total of 15/93 (16.13%) isolates were resistant to INH, 18/93 (19.35%) were resistant to RMP, and 11/93 (11.83%) of these isolates were resistant to both INH and RMP (MDR-TB). The mean turnaround time of culture and DST was 56.8 days (range, 49 to 77 days).

### Strain identification of *M. tuberculosis* using the DNA microarray

**Figure 3:**
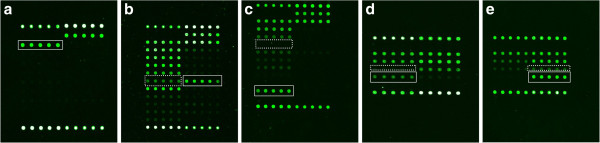
**Hybridization pattern of spinal tuberculosis specimens with the DNA microarray. a**. Hybridization pattern of the *M. tuberculosis* strain identification (white frame) **b**. RMP resistance with the *rpoB* 531 (TCG->TTG) mutation, the hybridization signal of probe TCG531TTG (solid rectangle) being higher than corresponding wild-type probe (dashed rectangle) **c**. RMP resistance test: *rpoB* 516 (GAC->GTC) mutation, the hybridization signal of probe GAC516GTC (solid rectangle) being higher than corresponding wild-type probe (dashed rectangle) **d**, **e**. INH resistance test: *katG* 315 (AGC->ACC) mutation, the hybridisation signal of probe *katG* AGC315ACC (solid rectangles) were higher than corresponding wild-type probe (dashed rectangles) e. INH resistance test: *inhA*-15 C-T mutation, the hybridisation signal of probe *inhA* 15C→T (solid rectangles) were higher than corresponding wild-type probe (dashed rectangles).

Among the 153 spinal tuberculosis specimens, 114 samples were identified to contain *M. tuberculosis* strains by the DNA microarray, and the positive detection rate was 74.51%. Among the 93 patients with culture-positive tuberculosis, the overall sensitivity of the *M. tuberculosis* identification by the DNA microarray was 93.55% (87/93). The sensitivity was 97.4% for smear- and culture-positive cases and 73.3% for smear-negative, culture-positive cases (Table 1). The hybridization pattern of the *M. tuberculosis* identification was shown in Figure 3a.

**Table 1:** **Overall sensitivity of the DNA microarray for the*M. tuberculosis*strains identification**

**Variable**	**Sensitivity**		
	**All Culture-Positive**	**Smear-Positive and Culture-Positive**	**Smear-Negative and Culture-Positive**
Correct-no./total no. (%)	87/93 (93.55)	76/78 (97.44)	11/15 (73.33)
95% CI	88.56-98.54	93.93-100.00	50.95-95.71

### Detection of the RMP and INH resistance using the DNA microarray

#### Detection of *M. tuberculosis rpoB* mutants

Taking the results of the phenotypic DST into account, the DNA microarray correctly detected *rpoB* mutations in 16 of the 18 patients with RMP resistance (88.89% sensitivity). The RMP-resistant samples displayed different mutations: 56.3% (9/16) of the samples had mutations at codon 531, 25.0% (4/16) had mutations at codon 526, and 18.8% (3/16) had mutations at codon 516. Additionally, one of the 18 (5.6%) RMP-resistant specimens produced the wild-type hybridization pattern on the DNA microarray, repeat testing and sequencing also revealed the wild-type pattern. Moreover, one of the 18 specimens (5.6%) failed to produce the hybridization pattern. Part of the hybridization patterns for the RMP-resistant samples was shown in Figure 3b, c. Two of the 75 (2.7%) RMP-susceptible specimens showed mutations at codon 533 (CTG->CCG), and one specimen showed a mutation at codon 513 (CAA->CCA). Four specimens (5.3%) failed to produce the pattern of the mutation. Regarding RMP resistance detection, the sensitivity and specificity of the DNA microarray were 88.9% and 90.7%, respectively (Table 2).

**Table 2:** Performance of the DNA microarray assay for detection of resistance to INH and RMP compared with phenotypic DST

**Phenotypic DST**	**DNA microarray assay**			
	**Sensitivity**		**Specificity**	
	**Correct-no./total no. (%)**	**95% CI**	**Correct-no./total no. (%)**	**95% CI**
RMP resistance	16/18 (88.89)	74.37-100.00	68/75 (90.67)	84.08-97.25
INH resistance	11/15 (73.33)	50.95-95.71	71/78 (91.03)	84.68-97.37

#### Detection of *M. tuberculosis inhA* and *katG* mutants

Of the 15 INH-resistant specimens, 8 specimens (53.3%) had a mutation in *katG* (codon 315), which is the most common mutation found in INH-resistant strains. Three of the specimens (20.0%) had a mutation in the *inhA* gene, C15T. Additionally, two low-level INH-resistant specimens (13.3%) displayed the wild-type hybridization pattern on the DNA microarray, and 2 of the specimens (13.3%) failed to produce the hybridization pattern. Part of the hybridization pattern of the INH-resistant isolates was shown in Figure 3d, e. Moreover, three of the 78 INH-susceptible samples (3.9%) showed a mutation at nucleotide 15 within the promoter of the *inhA*, and 4 of the 78 INH-susceptible samples (5.1%) failed to produce and results. The sensitivity and specificity of the INH resistance detection by the DNA microarray were 80.0% and 91.0%, respectively (Table 2). The mean turnaround time of *M. tuberculosis* identification and drug-resistance detection using DNA microarrays was 5.8 hours (range, 4 to 9 hours).

## Discussion

To respond to the urgent need for rapid and accurate detection of *M. tuberculosis* and drug resistance, a DNA microarray system was developed by the CapitalBio Corporation. This microarray can simultaneously identify 17 mycobacterial species by targeting the species-specific sequences in the 16S rRNA. Moreover, the specialized microarray consists of 16 oligonucleotide probes, which allowed the detection of the 13 most common mutations in the *rpoB* gene, 2 mutations in the *katG* gene, and one mutation in the promoter region of the *inhA* gene. In our study, the identification of *M. tuberculosis* strains and detection of their resistant form in spinal tuberculosis specimens were evaluated by the CapitalBio™ DNA microarray assay.

We evaluated the utility of the CapitalBio™ DNA microarray in 153 consecutive spinal tuberculosis samples. The microarray system identified more than 93% of all cases with culture-confirmed spinal tuberculosis, including more than 73% of patients with smear-negative cultures. Moreover, this assay was positive in 45% (27/60) of the patients who were not confirmed by *M. tuberculosis* culture but were demonstrated both clinically and pathologically to have spinal tuberculosis. Results of the study revealed that the microarray assay has a higher susceptibility compared with the conventional BACT/MGIT 960 system for the detection of *M. tuberculosis* strains. Previous studies conducted by Guo and Zhu *et al.* also reported that for the direct detection of *M. tuberculosis* complex bacteria the overall sensitivity obtained with this commercial DNA microarray were 100% [11,12]. The negative control of the species identification has not been setup considering the following reasons: First, Guo and Zhu respectively found that the specificity of the CapitalBio™ DNA microarray was 100% for the species identification of *M. tuberculosis*[11,12]. Second, recruitment and enrollment of pyogenic spondylitis patients, with similar clinical characteristics and clinical specimens to spinal tuberculosis, are relatively difficult in our department. However, absence of the negative control might be a potential limitation of this study.

For the detection of anti-tuberculosis drug resistance, the DNA microarray had a sensitivity of 73.3% and specificity of 91.0% for INH resistance, and a sensitivity of 88.9% and specificity of 90.7% for RMP resistance. Interestingly, 5.6% of phenotypically defined RMP-resistant specimens and 13.3% of INH- resistant specimens had no mutations in related genes, suggesting that other mechanisms or mutations in other codons of the related genes may be responsible for the emergence of RMP and INH resistance. Moreover, a small proportion of specimens failed to produce results; one possible reason for the lack of results may be because of the exiguous *M. tuberculosis* bacilli contained in some specimens, or the DNA amplification inhibitors in the clinical specimens, resulting in a lack of DNA being extracted from these samples. Results of the DNA microarray assay could be obtained within a mean turnaround time of 5.8 hours from the start of the analysis.

Recently, some novel molecular DST methods based on nucleic acid amplification have been available. Among them, the Xpert MTB/RIF assay was endorsed by the WHO as a replacement for sputum smear microscopy [7,8,14-16]. Compared with the Xpert MTB/RIF assay, the CapitalBio™ microarray system has some limitations, e.g., the assay is semi-automatic, requires more experienced technical skills for manual manipulation and has more opportunities to create biohazard intermediates. However, the microarray system has two advantages compared to the Xpert MTB/RIF assays. First, the microarray assay is less costly because cartridges of the Xpert MTB/RIF are disposable. Second, the microarray assay can detect the gene mutations associated to both RMP and INH.

In addition, compared with isolates and sputum samples, the application of the DNA microarray in spinal tuberculosis samples is far more complex because of the diversity of clinical sample types, difficulties in obtaining adequate tissue for analyses and the methods of processing samples prior to analysis [14].

## Conclusions

Overall, the CapitalBio™ DNA microarray is a simple and accurate tool for *M. tuberculosis* identification and for the diagnosis of RMP and INH resistance, demonstrating that the system is likely to be applicable to spinal tuberculosis specimens. The automated readout and short turnaround time make this assay suitable for testing and decreases delays in diagnosis, without the need to build large numbers of advanced biosafety facilities. Nevertheless, the infrastructure and trained personnel required for this system are still not available except in a limited number of reference laboratories, which dramatically reduces its clinical utility in poverty-stricken zones. Thus, the integral and fully automatic DNA microarray system should be developed, which would have direct benefits to workflows and to biosafety in resource-scarce settings. The gene mutations that relate to other first-line and second-line drug resistance should be considered for incorporation into the further DNA microarray system because of the dissemination of second-line drug-resistant *M. tuberculosis* strains. Additional large-scale studies are needed to more further evaluate its diagnostic performance in spinal tuberculosis.

## Competing interests

The authors declare that they have no competing interests.

## Authors’ contributions

LL, ZZ and JX were the primary researchers, conceived the study, designed, participated in sample collection, performed laboratory experiments, conducted data analysis and drafted the manuscript for publication. FL, ZW, PC and MZ participated in doing the laboratory experiments, interpreting the results. FW, TH and JX reviewed the initial and final drafts of the manuscript. All authors read and approved the final manuscript.

## Pre-publication history

The pre-publication history for this paper can be accessed here:

http://www.biomedcentral.com/1471-2334/12/303/prepub
